# Malignant Endocarditis Treated by Antistreptococcic Serum

**Published:** 1906-05-26

**Authors:** 


### MALIGNANT ENDOCARDITIS TREATED BY ANTISTREPTOCOCCIC SERUM.

Dr. Nathan Raw [1] reports three cases of malignant endocarditis in two of which he obtained good results from the use of antistreptococcic serum. He insists upon the advisability of selecting a polyvalent serum and has himself employed one prepared under the direction of Dr. Dowson in the laboratories of Messrs. Burroughs, Wellcome and Co. The reason for such selection is that streptococci are not all of the same strain, and that a serum obtained from one strain and effective against this variety of micro-organism may be useless against other varieties. This is frequently illustrated in reports which chronicle failure with a serum from one laboratory followed by success with serum from a different source. In a polyvalent serum an attempt is made to obtain an agent effective against many varieties of streptococci, and thus to increase the chances that a satisfactory result will be secured. Dr. Raw also advocates the administration of the serum per rectum instead of by the hypodermic method as is more usual. The latter is painful and is apt to be followed by skin rashes and joint affections. On the other hand, when given by the bowel, equally good results are obtained with little or no chance of the disadvantages just mentioned. The procedure advised is as follows: The bowels are freely cleared by aperients and the rectum gently washed out with a little warm saline solution. The formula for injection is antistreptococcic serum 20 cc., normal saline solution at 100° F. 40 cc., and this is slowly introduced into the rectum night and morning, or as required. In the two successful cases reported by Dr. Raw the patient was in each instance a young woman with cardiac disease of several years' standing. There were the usual clinical phenomena of malignant endocarditis, including rigors, pyrexia, sweating, petechiæ, etc. In one case the serum was used for twenty days, a total of 560 cc. being administered; in the second case 580 cc. were employed. The unsuccessful case was one of acute puerperal septicæmia with endocarditis developing after admission to hospital.

The value of antistreptococcic serum in malignant endocarditis has other witnesses. Dr. Wm. Murrell [2] used it successfully in a case in which what appeared to be an ordinary rheumatic endocarditis developed into one of the malignant type. Pyrexia had continued for some five weeks before the serum was used, and whilst the first brand of serum produced no effect, a second preparation promptly brought the temperature down to normal. A remarkable case enforcing some important lessons in connection with malignant endocarditis has been recorded by Dr. Newton Pitt.[3] The patient was a boy of thirteen years who, after an attack of chorea, developed a loud cardiac murmur in association with rigors, pyrexia, albuminuria, and, presumably as the result of embolism, right hemiplegia. He became much emaciated and for some eight weeks was so ill that the hope of recovery was practically abandoned. Then antistreptococcic serum was given and was continued on alternate days for fully six weeks, at the end of which time the boy had so greatly improved that he was shortly able to leave the hospital "the picture of health." The case illustrates what is not always remembered—namely, that malignant endocarditis is a disease which may be prolonged over many months. The fact that in a case which had lasted for at least eight weeks recovery should follow the use of antistreptococcic serum is a powerful advertisement of the value of this method of treatment. Another fact of great clinical importance in the diagnosis of malignant endocarditis is the possibility that the physical signs of cardiac disease may be relatively inconspicuous. No doubt in some instances loud murmurs, or fresh murmurs developing under observation, may be appreciated, and when this is the case such facts, taken along with pyrexia and other constitutional changes, are of great significance; but this is by no means always the case. There may be no murmur at all, or only one that can be accounted for by the depreciated quality of the blood or by some leakage at the mitral orifice as a result of imperfect nutrition of the cardiac muscle. Hence the importance of such a case as that put on record by Sir Dyce Duckworth.[4] Here a boy of fourteen years with high temperatures and other evidences of general toxæmia died from malignant endocarditis affecting the mitral valve, and yet during his illness, which extended over a fortnight, a "slight murmur" was heard only on two occasions. The inference is obvious, that among the possible interpretations of more or less prolonged pyrexia, malignant endocarditis must be considered even in the absence of conclusive evidence of valvular disease, and that the most suggestive signs of such disease may be found, not on examination of the precordial region, but in facts such as an enlargement of the spleen, hæmaturia, and other clinical events, which are capable of interpretation as results of embolism. That malignant endocarditis is occasionally overlooked there can be little doubt. The diagnostic position is often one of great difficulty. The disease may be concealed under the suspicion of malaria, or one or other of its results, such, for example, as pleural effusion, swollen and painful joints, albuminuria, etc., may be regarded as a primary diagnosis and the essential condition on which these depend thus overlooked. It is all the more urgent that such a mistake should not now occur, seeing that a properly prepared antistreptococcic serum is often strikingly successful, and that this can be efficiently given per rectum, thus avoiding the pain and annoyance of the hypodermic method. It may be noted that these more recent cases are not the only ones which support the statement that the serum may be administered by the bowel. Possibly there are other records, but certainly one case was treated in this way by Sir Dyce Duckworth [5] in 1902. The patient was a lad of fifteen years, and after various medicines had been tried without avail for some five or six weeks, a dose of 10 cc. of a mixed antistreptococcic serum was given per rectum and was repeated daily for many weeks. The pyrexia was subdued within two weeks after the rectal injections had been commenced, and in the end the boy completely recovered. Here again is illustrated not only the value of the serum and the efficiency of the introduction through the bowel, but also the necessity for repeating the dose of the serum at relatively frequent intervals. Somehow or other a notion has arisen that if one or two doses of the serum produce no decided change it is useless to continue it. Such a result may be a reason for trying a new brand of serum, but not for renouncing the line of practice. But, as already explained, a polyvalent serum gives a more secure confidence, as it is efficacious not merely against one, but against several varieties of organisms.

[1] Lancet, April 21, 1906. [2] Medical Press, April 10, 1905. [3] Lancet, Nov. 12, 1904. [4] Brit. Med. Jour., July 2, 1904. [5] Ibid, March 23, 1903.

